# Molecular Dynamics Simulations of Wild Type and Mutants of SAPAP in Complexed with Shank3

**DOI:** 10.3390/ijms20010224

**Published:** 2019-01-08

**Authors:** Lianhua Piao, Zhou Chen, Qiuye Li, Ranran Liu, Wei Song, Ren Kong, Shan Chang

**Affiliations:** 1 Institute of Bioinformatics and Medical Engineering, School of Electrical and Information Engineering, Jiangsu University of Technology, Changzhou 213001, China; lianhuapiao@jsut.edu.cn (L.P.); chenzhou@jsut.edu.cn (Z.C.); youer99311216@jsut.edu.cn (Q.L.); lrr@jsut.edu.cn (R.L.); dxxsw@jsut.edu.cn (W.S.); 2 School of Chemical and Environmental Engineering, Jiangsu University of Technology, Changzhou 213001, China; 3 School of Automobile and Traffic Engineering, Jiangsu University of Technology, Changzhou 213001, China

**Keywords:** Shank3 and SAPAP interactions, molecular dynamics simulation, free energy calculation, conformational change

## Abstract

Specific interactions between scaffold protein SH3 and multiple ankyrin repeat domains protein 3 (Shank3) and synapse-associated protein 90/postsynaptic density-95–associated protein (SAPAP) are essential for excitatory synapse development and plasticity. In a bunch of human neurological diseases, mutations on Shank3 or SAPAP are detected. To investigate the dynamical and thermodynamic properties of the specific binding between the N-terminal extended PDZ (Post-synaptic density-95/Discs large/Zonaoccludens-1) domain (N-PDZ) of Shank3 and the extended PDZ binding motif (E-PBM) of SAPAP, molecular dynamics simulation approaches were used to study the complex of N-PDZ with wild type and mutated E-PBM peptides. To compare with the experimental data, _974_QTRL_977_ and _966_IEIYI_970_ of E-PBM peptide were mutated to prolines to obtain the M4P and M5P system, respectively. Conformational analysis shows that the canonical PDZ domain is stable while the βN extension presents high flexibility in all systems, especially for M5P. The high flexibility of βN extension seems to set up a barrier for the non-specific binding in this area and provide the basis for specific molecular recognition between Shank3 and SAPAP. The wild type E-PBM tightly binds to N-PDZ during the simulation while loss of binding is observed in different segments of the mutated E-PBM peptides. Energy decomposition and hydrogen bonds analysis show that M4P mutations only disrupt the interactions with canonical PDZ domain, but the interactions with βN1′ remain. In M5P system, although the interactions with βN1′ are abolished, the binding between peptide and the canonical PDZ domain is not affected. The results indicate that the interactions in the two-binding site, the canonical PDZ domain and the βN1′ extension, contribute to the binding between E-PBM and N-PDZ independently. The binding free energies calculated by MM/GBSA (Molecular Mechanics/Generalized Born Surface Area) are in agreement with the experimental binding affinities. Most of the residues on E-PBM contribute considerably favorable energies to the binding except A963 and D964 in the N-terminal. The study provides information to understand the molecular basis of specific binding between Shank3 and SAPAP, as well as clues for design of peptide inhibitors.

## 1. Introduction

Scaffold proteins are highly abundant in dendritic spine and synapses. Specific interactions between the scaffold proteins play critical roles in the dendritic spine and synapse formation, maturation and maintenance [1]. SH3 and multiple ankyrin repeat domains protein 3 (Shank3) is one of the major scaffold proteins belonging to a family of higher order organizing molecules of the postsynaptic density and is involved in human neurological diseases [2,3]. The gene deletions of Shank3 cause the development of 22q13 deletion syndrome (Phelan-Mcdermid syndrome) and autism spectrum disorders (ASD) [4,5,6,7]. The disruption of the Shank3 gene in mice models directly leads to the genesis of the autistic-like behaviors [8,9]. Besides, the overexpression of Shank3 leads to hyperkinetic neuropsychiatric disorder [10]. A proper dosage of Shank3 is critical for normal brain function.

Although a bunch of proteins have been reported to bind to the PDZ (Post-synaptic density-95/Discs large/Zonaoccludens-1) domain of Shank3, the binding affinity of SAPAP (synapse-associated protein 90/postsynaptic density-95–associated protein; also known as GKAP or DLGAP) with Shank3 is about 100-fold stronger than the other reported binding proteins, and thus SAPAP is identified as the specific binding partner of Shank3 [11]. The dosage changes of Shank3 would have a major impact on the concentration of the Shank3/SAPAP complex instead of other reported Shank3 complexes due to the specific and high affinity binding between the two proteins. Indeed, the human nervous systems are highly sensitive to the dosages of these two proteins, meaning that a 50% decrease caused by a deletion/null mutation or a 50% increase due to gene duplications of Shank3 or SAPAP can cause disease [12]. Moreover, the behavioral abnormalities caused by mutations of SAPAP are significantly overlapped with those caused by Shank3 mutations. For example, loss-of-function mutations of SAPAP also lead to ASD-like phenotypes in both patients and animal models [13]. All the facts show that the interactions between Shank3 and SAPAP are critical for the normal physiological functions of synapses.

**Figure 1:**
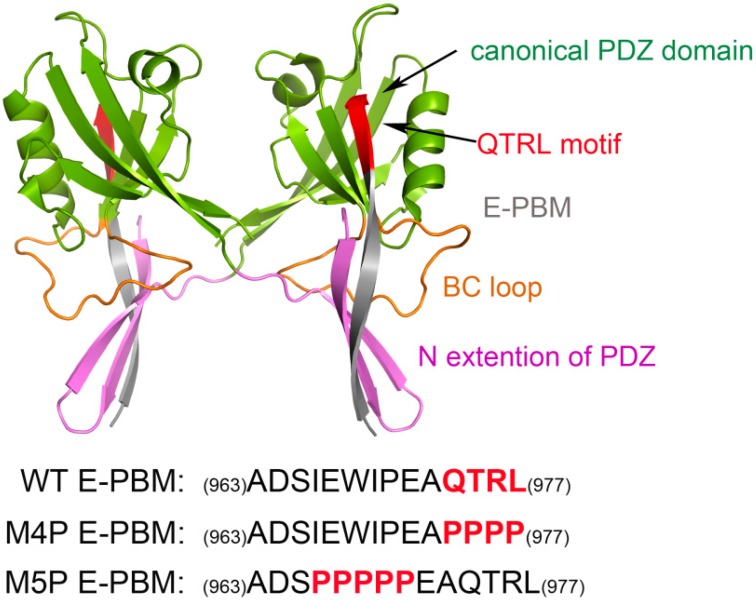
Shank 3-SAPAP modeling system. Protein is shown in ribbon model and the domains are colored according to the text in the map. The sequences of wild type E-PBM and mutated peptides are listed on the bottom.

Structural biology studies have reported that a canonical PDZ binding pocket of Shank protein interacts with the carboxyl terminus peptide from its ligands via the loosely defined binding motif X-(Ser/Thr)-X-Φ-COOH (Φ represents hydrophobic residues) [14,15,16]. The C-terminal binding peptides are named as PDZ binding motif (PBM). Due to the extraordinary high binding affinity between Shank3 and SAPAP, distinguishing structural characteristics may exist between the two proteins. Binding affinity measurements for various lengths of Shank3 and SAPAP showed that the Shank3 PDZ domain with an N-terminal extended β hairpin (residues 533–665, denoted “N-PDZ” for the N-terminal extended PDZ domain) and the last 12 residues of SAPAP3 (residues 966–977, referred to as “E-PBM” for the extended PDZ binding motif) are the minimal sequences for the strong interaction between Shank3 and SAPAP3. The recently reported crystal structure of Shank3 with SAPAP E-PBM showed that N-PDZ was separated to two sub-domains and referred to as the canonical PDZ domain (564–665) and the N extension of PDZ (542–563) (Figure 1). It is disclosed that in addition to the interactions of QTRL motif with the canonical PDZ binding pocket, the extensional residues from PBM region form interactions with N-extension β hairpin (βN hairpin) in Shank3 [11]. Specifically, two Shank3 N-PDZ chains form a swapped dimer, and the C terminal residues from E-PBM (QTRL motif) locate in the PDZ binding pocket from one monomer, and the N terminal residues of E-PBM contact with the βN hairpin from the other monomer (Figure 1). The biochemistry and structural experiments provide fundamental knowledge basis for Shank3 and SAPAP binding. However, several issues still remain unclear. How does the additional binding site affect the specific interaction of proteins? Why do the different mutants reduce the binding affinities? What conformational changes will happen in the wild and mutated systems?

In order to explore the above issues, molecular simulation methods are engaged to explore the binding of N-PDZ with the wild type and mutated E-PBM peptides. Theoretical modellings nicely complement the experimental techniques by providing valuable insight into protein dynamics characteristic at atomic level [17,18,19]. Basdevant et al. used molecular dynamics (MD) simulation method to study the binding of PDZ domain with various ligands. The promiscuity and selectivity in protein-protein interactions were explained by using the different dynamical properties of the ligands [20]. Here, we used full-atom MD simulation to investigate the molecular mechanism of the binding between Shank3 with wild type SAPAP E-PBM as well as two mutated peptides. To compare with the experimental data, residues of _974_QTRL_977_ on C-terminal and residues of _966_IEIYI_970_ on N-terminal of E-PBM peptide were mutated to prolines to obtain the M4P and M5P system, respectively. The dynamical properties such as conformational changes caused by the mutations were explored by using the dynamical cross-correlated map (DCCM) analysis, principal component analysis (PCA) and free energy landscape (FEL) methods. Molecular Mechanics/Generalized Born Surface Area (MM/GBSA) method was used to evaluate the binding free energy. We analyzed the crucial interactions between Shank3 and SAPAP E-PBM, and explained the loss of binding affinities of the two mutants relative to the wild type.

## 2. Results

### 2.1. Convergence of the Systems

**Figure 2:**
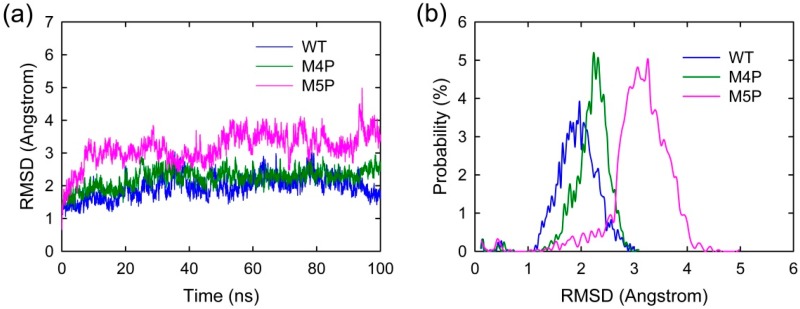
(**a**) RMSD evolutions of protein Cα atoms with simulation time in WT, M4P and M5P systems; (**b**) Probability distribution of RMSD values.

MD simulations were carried out separately for 100 ns on three systems, the wild type system and two mutated systems. The crystal structure presents an axisymmetric topology with two Shank3 N-PDZ chains in the center and the E-PBM peptide bound to the canonical PDZ domain from one monomer and βN hairpin from the other monomer (Figure 1). To investigate the contribution of different segments of E-PBM peptide to the binding and compare to the experimental data, 974–977 (QTRL) and 966–970 (IEIWI) were mutated to four and five prolines as shown in Zeng’s paper [11]. The wild type system and the two mutated systems are referred to as WT, M4P and M5P system in the following text. The root mean square deviation (RMSD) was monitored during the simulation time to investigate the protein stability. All the RMSD values in Figure 2 were calculated by using crystal structure as reference and protein Cα atoms for least fitting. The RMSD values converged after 20 ns simulations in all the systems, thus the MD trajectories from 20–100 ns were chosen for the further conformational analyses (Figure 2a). The mean values of RMSD of the WT, M4P and M5P system were 1.94, 2.21 and 3.13 Å, respectively. As the proteins in the WT system were taken from the crystal structure and represented the equilibrium conformation, it is not surprise that the WT system had the lowest RMSD values compared to the two mutated systems. M4P and M5P were constructed based on WT structure and the mutations may introduce the perturbations and conformation rearrangements to the structure, reflected by the increased RMSD values in the two systems. Especially for M5P system, the RMSD values showed continuous increase and the RMSD distribution shifted to the left axes with largest distance (Figure 2a,b).

**Figure 3:**
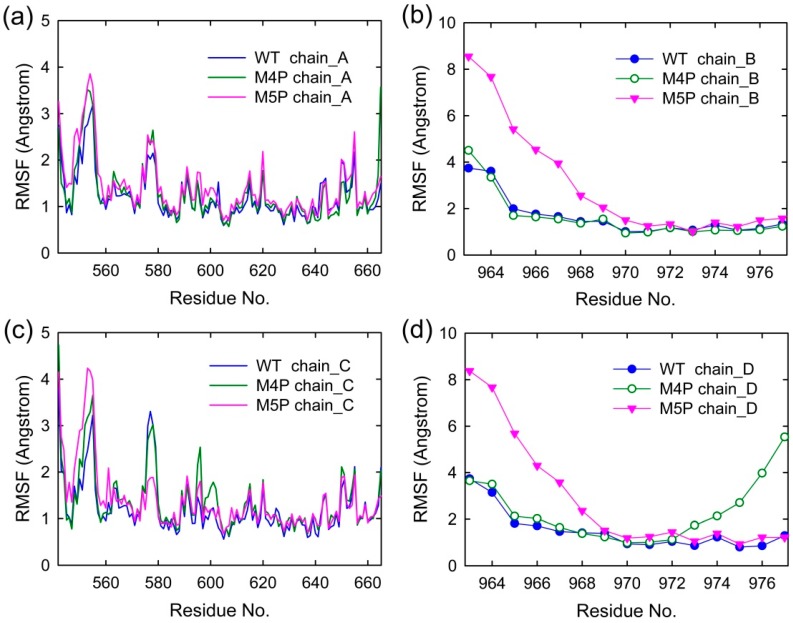
Per residue RMSF of four chains in WT, M4P and M5P systems. (**a**) RMSF of N-PDZ (chain A); (**b**) RMSF of E-PBM (chain B); (**c**) RMSF of N-PDZ (chain C); (**d**) RMSF of E-PBM (chain D).

Figure 3 shows the root mean square fluctuations (RMSF) of all the systems, indicating the amplitude of atom displacements. In the WT system, most of the regions in the canonical PDZ domain were with low RMSF except several parts: the loop connecting βA and βB (578–582), the loop connecting βF and αB (652–656), and αA (615–622) (Figure 3a,c). The flexible parts of canonical PDZ domain probably provided the molecular basis to enable the reconfiguration of binding pocket and accommodate the binding of different ligands [14]. Part of βN hairpin (548–556) in N extension of PDZ possessed relatively large RMSF values, indicating the high flexibility in this area. The E-PBM peptides in WT system possessed low RMSF values less than 2 Å except two N terminal residues, 963 and 964 (Figure 3b,d). The relatively high RMSF of N terminal residues of E-PBM may be caused by the large movements of its binding partner, βN hairpin.

In M4P system, one of the E-PBM chains showed large RMSF values on the C-terminal residues 973–977, indicating the mutation of C-terminal residues 974–977 (QTRL) to prolines disabled the tight binding in the canonical PDZ domain (Figure 3d). The residues located in BC loop (592–602) of one monomer in the M4P system possessed the highest RMSF values compared to the other two systems (Figure 3c). It seems the mutations also increased the flexibility of BC loop. However, the RMSF values of other residues of E-PBM in M4P systems were similar to those in the WT system.

The residues of 548–556 located on βN hairpin possessed the highest RMSF values in the M5P system (Figure 3a,c). Correspondingly, remarkable increases in RMSF values were observed for residues 963 to 968 of the mutated E-PBM peptides (Figure 3b,d). It seems the mutations of IEIWI (966–970) to five prolines introduced extraordinarily high flexibility to the N terminal extending sequence of E-PBM and its binding part βN hairpin. However, the RMSF values of the E-PBM C terminal residues in M5P system were as low as those in the WT system, especially for the QTRL parts. It seems the mutations only affected the binding of the N terminal part of E-PBM to βN hairpin, not the binding of C terminal residues to the canonical PDZ domain. Binding affinities measured by the ITC-based experiments showed that the IEIWI (966–970) to prolines weakened the binding between shank N-PDZ and SAPAP E-PBM to approximately five-fold, a value comparable to the ligands only binding to the canonical PDZ domain [11]. The modeling observations were well consistent with the experimental data. In addition, to avoid any random conclusions, we conduct duplicate MD simulations for all the systems. The RMSD and RMSF distributions of the duplicate trajectories were similar to the original ones, implying the intrinsic conformational changes caused by the mutations (Supplementary Figures S1 and S2).

### 2.2. Dynamical Cross-Correlated Map Analysis for Three Systems

**Figure 4:**
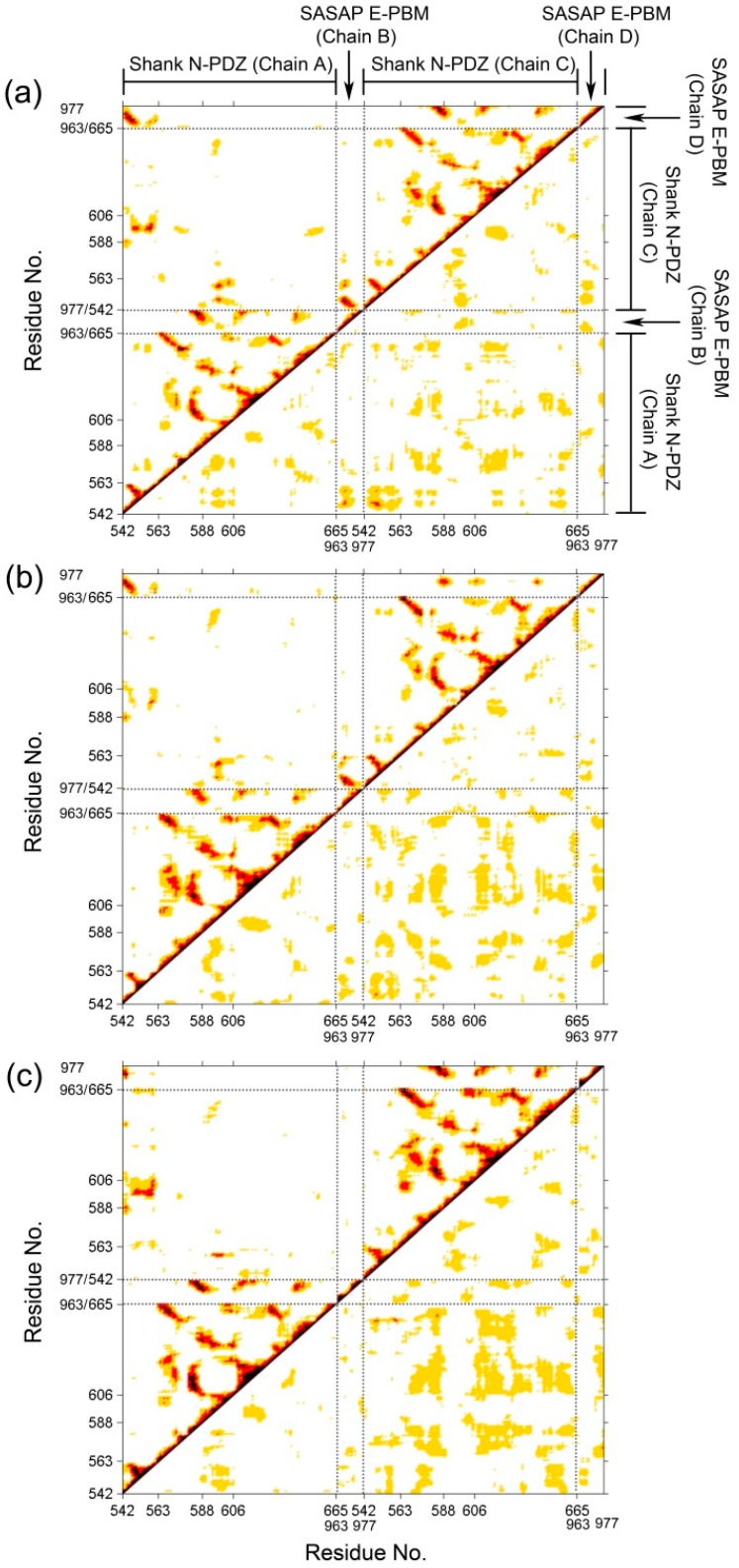
Dynamical cross-correlated map (DCCM) of WT (**a**), M4P (**b**) and M5P (**c**) systems. Deeper color indicates more correlated or anti-correlated. Both x and y axes are residue indices of proteins.

To investigate the correlative motions of Shank3 and SAPAP, dynamical cross-correlated maps (DCCM) were generated. The correlated fluctuations of Shank N-PDZ and SAPAP E-PBM in WT, M4P and M5P systems are graphically presented in Figure 4. Positive correlations are mapped in the upper left triangle while negative correlations are mapped in the lower right triangle. Deeper color indicates more positive or negative correlated motions between the structural patterns. In the map of the WT system (Figure 4a), E-PBM showed strong positive correlations with βN1′ (542–549), βB (583–588), βC (606–613), αB (642–649). The residues from these regions were found to form hydrogen bonds, salt bridges or hydrophobic interactions with E-PBM peptide in the crystal structures [11]. The strong positive correlation motion modes reflected tight binding of E-PBM with βN hairpin and the canonical PDZ pocket residues. In the M4P system, although the positive correlations remained between E-PBM and βN1′, the correlations between E-PBM with the canonical PDZ residues decreased a lot, especially in chain D (Figure 4b). It implies the mutations weakened the interactions between E-PBM and the canonical PDZ domain. Weak binding was detected between the QTRL deleted E-PBM and Shank3 SH3-PDZ with N extension domain, consistent with the remained binding to βN hairpin in M4P system [11]. In the M5P system, the positive correlations between E-PBM and βN1′ weakened a lot while those between E-PBM and canonical PDZ domain stayed the same. It seems the mutations in M5P only affected the binding between E-PBM and the N extension of PDZ, not the binding in the canonical PDZ domain pocket. Binding affinity measurements also showed that the K_d_ value of the M5P mutation was comparable with the typical value of the canonical PDZ and PBM interactions [11,21]. The DCCM results are consistent with the RMSF analysis and experimental data. Meanwhile, positive correlations were also observed between the BC loop (588–606) and βN hairpin (542–563) in the opposite monomer in all the systems except M4P. Spatially, the BC loop located nearby the βN hairpin from the other monomer (βN’ hairpin) and interactions were observed between them. The mutations in M4P seem to weaken the positive correlations between the BC loop from chain C and βN’ hairpin from chain A. By comparison of the maps, stronger negative correlations between the two N-PDZ chains were observed in the mutated systems, especially for the M5P systems. It indicates the enhanced relative movements between the two chains.

### 2.3. Motion Modes Analysis for WT, M4P and M5P System

**Figure 5:**
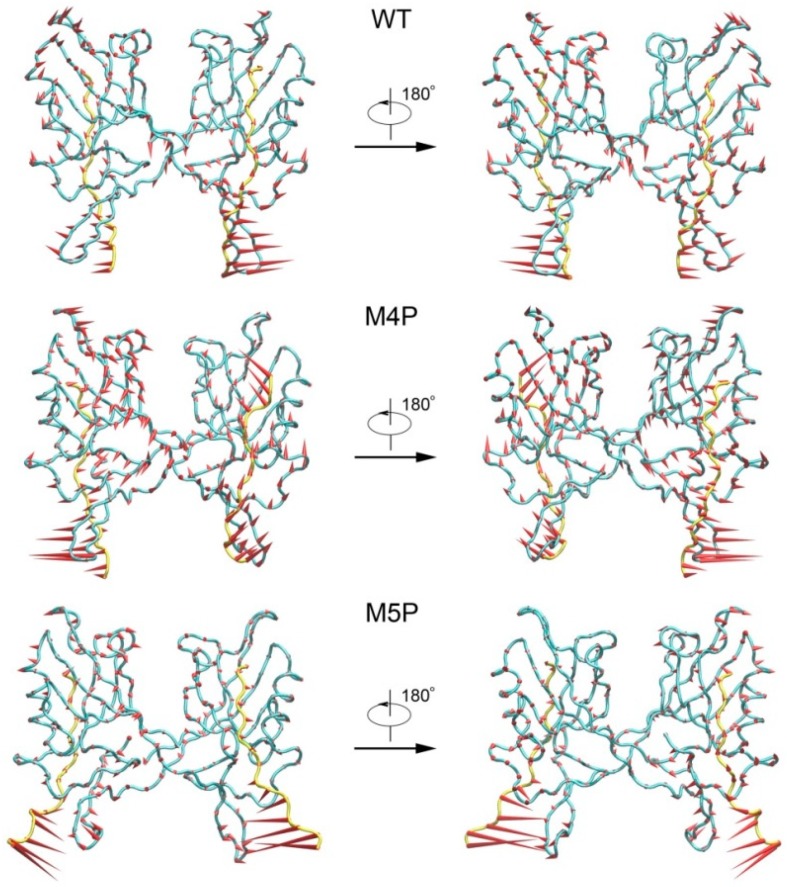
First slowest motion modes of WT, M4P and M5P systems. The protein Cα atoms are represented by tube model. Shank3 N-PDZ is colored in cyan and SAPAP E-PBM peptides are colored in yellow. The length of the cone is correlated positively with motion magnitude, and the orientation of the cone describes the motion direction.

PCA analyses were performed to extract the essential dynamical motions by filtering global slow motions from the fast motions for WT, M4P and M5P systems. The first principal movements (PC1) from PCA analysis is presented in Figure 5. The length of cone represents the motion magnitude and the pointing of the arrow shows the direction. In the WT system, the whole structure was stable except relatively large movements in βN hairpin and its binding part, N-terminal residues of E-PBM. The mutated C terminal residues possessed large movements in the M4P system, and it may induce the enhanced motions in the whole PDZ domain. The conformation of the N terminal part of E-PBM in M5P system was obviously deviated from that of the WT and M4P system. The amplitudes of motions of N-terminal residues on E-PBM and βN hairpin were large in all systems, but most significant in M5P system. Both parts moved towards the same direction in the WT and M4P systems but not in the M5P system. These results were in agreement with the DCCM analysis, which showed the abolishment of positive correlations between E-PBM and βN1′ only in M5P system. However, the motions of the canonical PDZ domain and C-terminal E-PBM in M5P system were relatively low, indicating low impact on the binding of this part.

**Figure 6:**
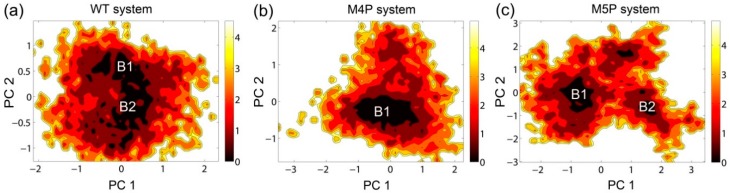
Free energy contour map depicted along the first and second principal components (PC1 & PC2) for WT (**a**), M4P (**b**) and M5P (**c**) systems. Deeper color area in the maps indicates lower energy. The distinctive local basins are denoted as B1 and B2, respectively.

The free energy contour maps were constructed for three systems at 298 K and shown in Figure 6. The deeper color indicates the lower energy. It is found that the PC1 and PC2 motion modes of the mutated systems spanned larger ranges than those of the WT system, indicating the conformational rearrangements caused by the mutations. Distinctive local basins from free energy landscapes were identified and representative structures were extracted and compared with the crystal structure (Figure 6, Supplementary Figure S3). As shown in Supplementary Figure S3, the QTRL residues in E-PBM moved away from the PDZ pocket in representative snapshot of the M4P system. Whereas in the M5P system, the N-terminal residues of E-PBM exhibited remarkable conformational changes and increased distances were observed between N-terminal residues of E-PBM and βN hairpin of N-PDZ. No obvious conformational changes in canonical PDZ domain were detected in all three systems.

### 2.4. Comparison the Interactions in WT, M4P and M5P Systems

To further explore the rearrangement of interactions in three systems, MM/GBSA free energy calculation and decomposition as well as hydrogen bonds were analyzed for these systems. The binding free energies and energy components are listed in Table 1. The ranking of calculated binding free energies is well consistent with that of experiment binding affinities [11]. The WT system had the lowest binding free energy and the M4P and M5P systems possessed much higher binding free energy. The electrostatic energy term (ΔE_electrostatic_) was counteracted by the electrostatic contribution solvation free energy (ΔG_GB_) and thus the electrostatic interactions contributed little to the total free energy. The van der Waals energy term (ΔE_vdw_) and the nonpolar solvation free energy (ΔG_SA_) were the major contributors to the binding free energy in all the systems. The tight binding of peptide in the WT system obtained lowest ΔE_vdw_. Whereas in the mutated system, different parts of E-PBM were observed to move away from the N-PDZ, which may cause the reduction of van der Waals energy contacts and the increase of binding free energy.

**Figure 7:**
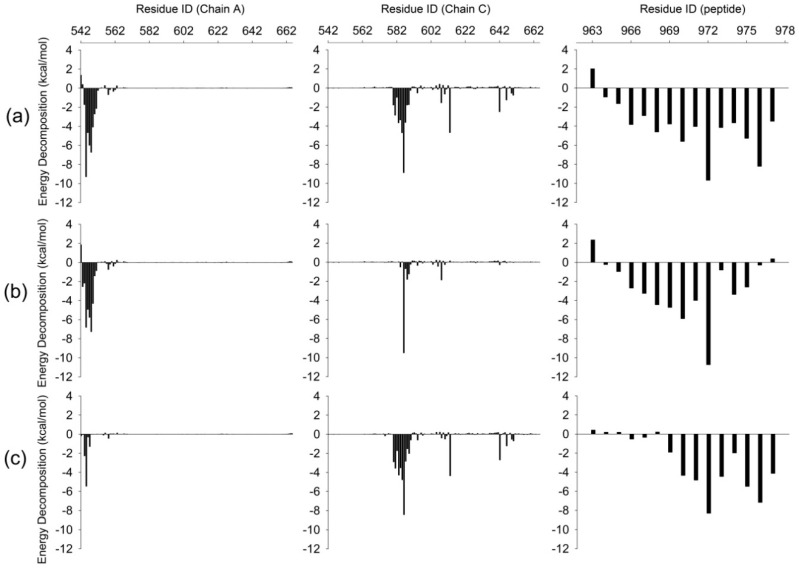
Binding free energy decomposition for WT (**a**), M4P (**b**) and M5P (**c**) systems. Energetic contributions of chain A and C in Shank N-PDZ dimer are shown in left and middle panel. Energetic contributions of SAPAP E-PBM are shown in left panel.

**Figure 8:**
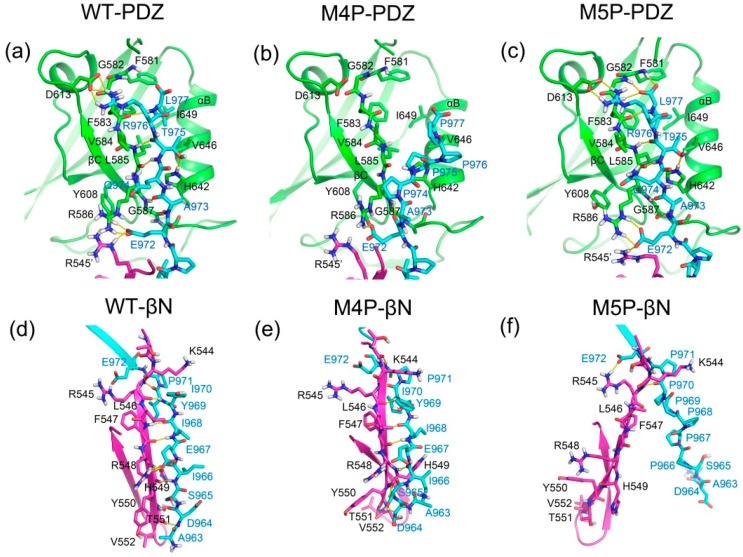
Representative binding modes from the MD simulation of WT, M4P and M4P systems. The binding of E-PBM in PDZ binding pocket are shown in (**a**) (WT, 47.36 ns), (**b**) (M4P, 66.84 ns) and (**c**) (M5P, 63.32 ns). The binding of E-PBM with βN1′ are shown in (**d**) (WT, 47.36 ns), (**e**) (M4P, 66.84 ns) and (**f**) (M5P, 63.32 ns). PDZ domain, βN1′ and E-PBM are colored in green, magenta and cyan, separately. Key residues are shown in stick model. Hydrogen bonds are shown in yellow dash lines.

To further investigate the detailed interactions between E-PBM and N-PDZ, energy decomposition strategy was used to analyze the energy contributions of each residue in three systems (Figure 7, Supplementary Table S1). Chain D was chosen as ligand while chain A and C composed of the dimer were chosen as receptor in the calculations. Spatially, chain D contacted with the βN hairpin extension from chain A and the canonical PDZ domain from chain C. The contributed residues in Figure 7 are mainly located in these regions. In the WT system, residues K544-T551 from βN1 sheet contributed favorable energy for the binding. In the canonical PDZ domain, residues G580-G587 from βB, residue A588 and K589 from BC loop, residues Y608 and D613 from βC, residues H642 and V646 from αB possessed energy contribution larger than 1 kcal/mol. These residues were found to form main chain or side chain hydrogen bonds or hydrophobic contacts with E-PBM in the crystal structure and the representative binding pose from the WT MD simulation (Figure 8a) [11]. With the mutations of QTRL to prolines, the energy contributions of residue 973–977 decreased dramatically in the M4P system (Figure 7b). Meanwhile, most of the energetically favorable residues in the canonical PDZ domain showed reduced or totally abolished energy contributions. But the contribution map of βN hairpin was similar to that of the WT system, indicating no obvious influence for the binding of this part (Figure 8b,e). On the contrary, the M5P mutations weakened the energy contribution of residues on the βN hairpin but did not affect the residues on the canonical PDZ domain (Figure 7c). In the representative pose from the trajectory of the M5P system, the N terminal residues of E-PBM moves away from βN hairpin and almost all hydrogen bonds with βN1′ disappeared (Figure 8f). However, the interactions in canonical PDZ domain remained quite well (Figure 8c). All the residues on E-PBM contributed favorable energy in the WT system except A963 and the energy contribution map of peptide may provide clues for structural optimization to improve the binding affinity (Table S1). The energy contributions of mutated residues reduced significantly in both M4P and M5P systems, which is in agreement with the RMSF and PCA analysis.

We further analyzed the hydrogen bonds occurrence between N-PDZ and E-PBM peptides. The hydrogen bonds formed between N-PDZ and E-PBM with occupancy over 30% for WT, M4P and M5P systems are listed in Table 2. In the canonical PDZ domain, β-strand pairing was formed between βB and E-PBM by a set of stable main chain hydrogen bonds between G587 and A973, L585 and T975, F593 and L977. The side chain of T975 hydrogen bonded to the side chain of H642 from αB and the side chain of R976 hydrogen bonded to the side chain of D613 from βC with remarkable high occupancy, indicating the stability of the interactions. These side chain hydrogen bonds were also reported in the structure of Shank1 and βPIX, and T975 is highly conserved in all the PDZ binding ligands [14]. P971 formed main chain hydrogen bonds with K589 from the BC loop and E972 formed hydrogen bonds to R545 from βN1′, R586 from βB, and Y608 from βC. The hydrogen bond network formed by P971 and E972 anchored the middle part peptide to the space between the PDZ and βN hairpin extension. The N terminal residues of E-PBM continuously formed β-strand pairing with βN1′ from N-PDZ in WT system and a set of stable main chain hydrogen bonds were found between residue pairs including F547-I968, H549-I966, R545-I970, T551-D964. Moreover, hydrogen bonds were also observed between the main chain of H549 and the side chain of S965. Most of the hydrogen bonds are conserved with those observed in the initial crystal structure [11]. The M4P mutations disrupted almost all the hydrogen bonds in canonical PDZ domain except the side chain hydrogen bonds with E972 (Table 2, Figure 8b,e). However, the majority of hydrogen bonds between βN1′ and E-PBM were retained. The M5P mutations kept only one native hydrogen bond in the βN1′ binding part, whereas all the native hydrogen bonds in the PDZ binding pocket were maintained as high occupancy hydrogen bonds, the same as in the WT system (Table 2, Figure 8c,f). The results imply that M4P and M5P mutations broke most of the native hydrogen bonds between E-PBM and the canonical PDZ domain or N-extension of PDZ (βN1′), respectively.

## 3. Discussion

In this study, we compared the binding of wild type and mutated E-PBM peptides to Shank3 N-PDZ by using molecular dynamics simulations. The M4P (_974_QTRL_977_ to four prolines) and M5P (_966_IEIYI_970_ to five prolines) mutations located on the C-terminal part and N-terminal part of E-PBM, respectively. The RMSF and PCA analysis showed the departure of mutated segments from its original binding positions. The DCCM analysis showed strong positively correlated motions between the wild type E-PBM with the canonical PDZ domain and βN1′ of N-PDZ. However, the positively correlated motions between E-PBM and the canonical PDZ domain were weakened in the M4P system while the correlated motions between E-PBM and βN1′ had disappeared in the M5P system. The results from conformational analysis imply that the mutations only destroyed the interactions with its corresponding binding partner, but have low impact to the other part of the peptide.

We also performed MD simulations on unbound E-PBM peptides (Supplementary Figures S4 and S5) to explore the conformational changes of peptides in free state. For wild type peptide, the β-strand conformation in bound state was not maintained in the free state and the C terminal residues tended to transform to helix. The mutations affected the secondary structure in free state. Both the wild type and mutated E-PBM peptides may conduct significant conformational changes upon the binding to N-PDZ.

The conformational changes on N-PDZ were monitored and it was found that no obvious conformational arrangements were detected in the canonical PDZ domain. But for the βN extension, relative high flexibility was observed in the wild type system and the flexibility was enhanced in the mutated systems, especially for M5P system. The canonical PDZ domain is the primary binding site of E-PBM and capable of binding to several proteins with PBMs [14,15,16]. The βN extension provides a secondary binding site outside the canonical PDZ domain and the additional binding between E-PBM and βN extension is believed to account for both the specificity and the enhanced binding affinity [11]. The high flexibility of βN extension seems to set up a barrier for the non-specific binding in this area and provide the basis for specific molecular recognition between Shank3 and SAPAP.

Free energy calculation and hydrogen bond analysis were employed to further quantitatively compare the interaction changes in wild types and mutated systems. It was shown that the M4P mutations caused the loss of most energy favorable contributed residues and almost all the hydrogen bonds in the canonical PDZ domain but the interactions with βN1′ from the N-extension of PDZ remained. The experimental data showed that the deletion of the QTRL motif completely eliminated the binding of E-PBM to the canonical Shank3 PDZ, but did not totally disrupt its binding to N-PDZ. However, the K_d_ values decreased dramatically from 0.022 to 63.3 μM [11]. The M5P mutations abolished the interactions of E-PBM with βN1′, but the interactions with the PDZ domain maintained quite well comparing to wild type peptide. The K_d_ values of M5P peptide decreased to 0.129 μM, a value comparable to the ligand only binding to canonical PDZ domain [11]. The simulation results are quite consistent with the experimental observations. The MM/GBSA binding free energies calculated based on the snapshots from MD simulations are also in agreement with the measured experimental binding affinities. The results indicate that the interactions in the two-binding site, the canonical PDZ domain and the βN1′ extension, contribute to the binding between E-PBM and N-PDZ independently. The inhibition of the binding of E-PBM to either of binding sites may lead to the decrease of the binding affinity. The energy decomposition of E-PBM showed that the N terminal A963 and D964 made unfavorable or low energy contributions to the binding. And E972 possessed the largest free energy contribution. Structurally E972 formed multiple hydrogen bonds with R545 from βN1′, R586 from βB, and Y608 from βC. E972 is conserved in SAPAP family and it may play as a key residue in the binding of E-PBM and Shank3 N-PDZ.

Shank3/SAPAP interactions play a fundamental role in synaptic activity. The Shank3 gene duplicate causes the hyperkinetic neuropsychiatric disorder and the patients present an undesired level of excitatory circuit activation [10]. E-PBM peptide has been proved to block the Shank/SAPAP complex formation and downregulate the synaptic activity in cell, which encourages the efforts to develop it into a potential drug for the diseases caused by the overexpression of Shank3 or SAPAP [11]. The simulation results provided the free energy contribution profile of each residue in E-PBM, which may benefit the peptide inhibitor optimization. Mutation or deletion of residues with unfavorable or weak energy contributions may increase the efficacy of the designed peptides.

## 4. Materials and Methods

### 4.1. Construction of Modeling Systems

The structure of Shank3 N-PDZ and SAPAP E-PBM was obtained from Protein Data Bank (PDB ID: 5IZU) [11]. To compare with the experimental data, two mutation systems were constructed by substituting the sequence of QTRL (974–977) and IEIWI (966–970) to prolines, separately (Figure 1). The bioluminate module from in Maestro 10.3 was used to construct the mutated E-PBM peptides [22]. The wild type and mutated systems were referred to as WT, M4P and M5P in the following text. Then, three independent molecular dynamics simulations were performed for the wild type and mutated systems.

### 4.2. Protocols of Molecular Dynamics Simulation

All MD simulations were carried out using AMBER16 [23] with ff14SB force field [24]. The proteins were put in a truncated octahedron periodic box with the nearest distance between the protein and the box boundary less than 10 Å. About 10,000 TIP3P water molecules were added in the solvation stage to obtain about 35,000 total atoms for the three systems. The SHAKE algorithm was applied to constrain all the covalent bonds with hydrogens [25]. The cutoff for Lennard-Jones interactions was set to 10 Å and the particle-mesh Ewald (PME) algorithm was used for the calculation of the electrostatic interactions [26]. 500 steps steepest-descent minimization followed by 1500 steps conjugated gradient minimization were applied to the systems. 200 ps NTV simulations were used to heat the systems to 300 K gradually with the solutes restrained by a weak harmonic potential. 400 ps NTP simulations were subsequently performed for equilibrations via two steps: 1. restrain the solutes and equilibrate the water and ions in the first 200 ps; 2. remove all the restraints in the last 200 ps. Finally, the production MD simulations were conducted at 1 atm and 300 K under the NPT ensemble for 100 ns. Temperature was controlled using Langevin dynamics [27]. The trajectory snapshots were saved every 2.0 ps, and 50,000 conformations were collected for further analyses. All images were generated by VMD 1.9.2 [28] or PyMol 1.5 [29].

### 4.3. Dynamic Cross-Correlation Map (DCCM)

To evaluate the dynamic correlations of domains, DCCM analysis was performed to compare the correlation matrix across all Cα atoms for all the systems. The correlation coefficient *S_ij_* between two atoms i and j during the course of the simulation trajectory is defined as:

(1) $$S_{ij}=\frac{\langle \Delta r_{i}\cdot \Delta r_{j}\rangle }{\sqrt{\langle \Delta r_{i}\cdot \Delta r_{i}\rangle \langle \Delta r_{j}\cdot \Delta r_{j}\rangle }}$$

where displacement vectors Δ*r_i_* or Δ*r_j_* are calculated by subtracting the instantaneous position of *i*th or *j*th atoms with its average position. *S_ij_* > 0 represents the positively correlated motions between the *i*th and *j*th atom and *S_ij_* < 0 represents the negatively correlated motions between them.

### 4.4. Principal Component Analysis (PCA) and Free Energy Landscape (FEL)

PCA was widely used to extract the slow and functional motions for bio-molecules [30]. To perform PCA, the covariance matrix *C* was calculated firstly. The elements *C_ij_* in the matrix *C* are defined as:

(2) $$C_{ij}=\langle \left(x_{i}-\langle x_{i}\rangle \right)\left(x_{j}-\langle x_{j}\rangle \right)\rangle$$

where $x_{i}$ and $x_{j}$ are the instant coordinates of the *i*th or *j*th atom, and $\langle x_{i}\rangle$ and $\langle x_{j}\rangle \;$ mean the average coordinate of the *i*th or *j*th atom over the ensemble. The principal components (PCs) are obtained by diagonalization and solving the eigenvalue and eigenvectors for the covariance matric *C*. The eigenvectors, PCs, represent the directions of the motions while the eigenvalues show the magnitude of the motions along the direction. The analysis tools, g_covar and g_anaeig from Gromacs 4.5.5 package [31] were used to perform the PCA analysis. Free energy landscape (FEL) could be performed to explore the conformation change of biomolecules based on the PCA [32,33,34]. According to the free energy values of the conformations, stable state and transient state of bio-molecules could be identified.

(3) $$\Delta G\left(X\right)=-K_{B}TInP\left(X\right)$$

The free energy, Δ*G*(*X*), is calculated by Equation (3), where $K_{B}$ is the Boltzmann constant and *T* is absolute temperature, *X* represents the PCs, and *P*(*X*) is the probability distribution of the conformation ensemble along the PCs.

### 4.5. MM/GBSA Binding Energy Calculation

Molecular Mechanics/Generalized Born Surface Area (MM/GBSA) has been widely used to calculate binding free energy for protein-ligand systems [35,36,37]. Here, the python script MMPBSA.py in Amber 16 was used to calculate the binding energy for the wild type and mutated systems. Totally 400 snapshots were extracted from the last 40 ns trajectory from production simulation for MMGBSA free energy calculation. Per residue energy decomposition was also performed to evaluate the energy contribution of each residue in the systems. All the other parameters are kept as default value.

### 4.6. Hydrogen Bond Analysis

All the hydrogen bonds were calculated by VMD 1.9.2 [28] with a distance cut-off value of 3.5 Å and an angle cut-off value of 35° [38].

## 5. Conclusions

In this work, MD simulations were applied to investigate the binding of wild type and mutated E-PBM peptides with Shank3 N-PDZ. The conformational analysis showed that for N-PDZ, the canonical PDZ domain was stable while the N extension β hairpin showed relatively large flexibility in all simulations. The high flexibility of the N extension β hairpin may provide a barrier for the non-specific binding in this area. For E-PBM, although the wild type peptide bound tightly to N-PDZ during the simulation, loss of binding was observed in different segments of the mutated peptides. Energy decomposition and hydrogen bonds analysis showed that the mutations on C-terminal _974_QTRL_977_ residues (M4P system) disrupted only the interactions with canonical PDZ domain, but the interactions with βN1′ remain. The mutations on the N-terminal _966_IEIYI_970_ residues (M5P system) abolished the interactions with βN1′, without affecting the binding with the canonical PDZ domain. The binding energies calculated by MM/GBSA for wild type and mutated peptides were in agreement with the experimentally measured binding affinities. The results indicate that the interactions in the two-binding site, the canonical PDZ domain and the βN1′ extension, contribute to the binding between E-PBM and N-PDZ independently. The additional binding between the flexible N extension β hairpin and E-PBM may provide the molecular basis for the specific recognition between Shank3 and SAPAP. Most of the residues on E-PBM present considerably favorable energies to the binding except A963 and D964 in the N-terminus of E-PBM. This study provides insights into the molecular mechanisms of the specific binding between scaffold protein Shank3 and SAPAP, as well as clues for the peptide inhibitors design to interfere with the interactions between them.

## Figures and Tables

**Table 1:** Comparison of binding energy components between Shank3 N-PDZ and SAPAP E-PBM in three complex systems.

Systems	ΔE_vdw_	ΔE_electrostatic_	ΔG_GB_	ΔG_SA_	ΔG_MMGBSA_
WT	−113.48 ± 6.78	−597.51 ± 42.78	590.25 ± 39.03	−16.83 ± 0.69	−137.57 ± 8.75
M4P	−87.49 ± 5.55	−503.86 ± 35.90	513.23 ± 33.36	−11.63 ± 0.72	−89.76 ± 6.46
M5P	−84.92 ± 7.86	−471.01 ± 34.92	472.84 ± 32.64	−12.58 ± 1.11	−95.68 ± 8.79

All the energies are in kcal/mol; 400 snapshots extracted from the last 40 ns MD simulation were submitted to MMPBSA.py for the free energy calculation. ΔE_vdw_: van der Waals interaction energy; ΔE_electrostatic_: electrostatic interaction energy; ΔG_GB_: electrostatic salvation energy (polar contribution); ΔG_SA_: non-electrostatic salvation component (non-polar contribution); ΔG_MMGBSA_: calculated binding free energy by Molecular Mechanics/Generalized Born Surface Area (MM/GBSA).

**Table 2:** The hydrogen bonds formed by Shank N-PDZ and SAPAP E-PBM with occupancy over 30% for WT, M4P and M5P system.

WT			M4P			M5P		
βN1′	E-PBM	Occupancy	βN1′	E-PBM	Occupancy	βN1′	E-PBM	Occupancy
PHE547-Main-O	ILE968-Main-N	94.86%	**PHE547-Main-O**	**ILE968-Main-N**	**94.30%**	ARG545-Main-N	PRO970-Main-O	89.76%
HIE549-Main-N	ILE966-Main-O	93.68%	**ARG545-Main-O**	**ILE970-Main-N**	**92.40%**	**ARG545-Side-NH1**	**GLU972-Side-OE1**	**52.44%**
ARG545-Main-O	ILE970-Main-N	92.42%	**ARG545-Main-N**	**ILE970-Main-O**	**89.02%**			
PHE547-Main-N	ILE968-Main-O	83.88%	THR543-Main-O	GLU972-Main-N	88.90%			
HIE549-Main-O	ILE966-Main-N	70.34%	**PHE547-Main-N**	**ILE968-Main-O**	**79.68%**			
ARG545-Main-N	ILE970-Main-O	68.04%	**HIE549-Main-N**	**ILE966-Main-O**	**67.28%**			
THR551-Main-N	ASP964-Main-O	46.18%	**ARG545-Side-NE**	**GLU972-Side-OE2**	**49.26%**			
ARG542-Side-NE	GLN974-Side-OE1	43.14%						
HIE549-Main-O	SER965-Side-OG	40.82%						
ARG545-Side-NE	GLU972-Side-OE1	37.84%						
THR551-Main-O	ASP964-Main-N	35.36%						
**BC loop**			**BC loop**			**BC loop**		
LYS589-Main-N	PRO971-Main-O	94.44%	**LYS589-Main-N**	**PRO971-Main-O**	**93.80%**	**LYS589-Main-N**	**PRO971-Main-O**	**95.02%**
**PDZ**			**PDZ**			**PDZ**		
LEU585-Main-O	THR975-Main-N	96.08%	**TYR608-Side-OH**	**GLU972-Side-OE2**	**89.62%**	**LEU585-Main-O**	**THR975-Main-N**	**96.68%**
GLY587-Main-N	ALA973-Main-O	90.46%	**ARG586-Side-NH2**	**GLU972-Side-OE1**	**81.12%**	**PHE583-Main-O**	**LEU977-Main-N**	**93.36%**
GLY587-Main-O	ALA973-Main-N	90.14%				**GLY587-Main-N**	**ALA973-Main-O**	**90.94%**
LEU585-Main-N	THR975-Main-O	86.48%				**HIE642-Side-NE2**	**THR975-Side-OG1**	**90.50%**
PHE583-Main-O	LEU977-Main-N	83.66%				**LEU585-Main-N**	**THR975-Main-O**	**89.40%**
HIE642-Side-NE2	THR975-Side-OG1	79.64%				**GLY587-Main-O**	**ALA973-Main-N**	**83.22%**
ASP613-Side-OD1	ARG976-Side-NH2	65.36%				**PHE581-Main-N**	**LEU977-Side-OXT**	**53.70%**
ASP613-Side-OD2	ARG976-Side-NH1	64.44%				**ASP613-Side-OD2**	**ARG976-Side-NH2**	**48.48%**
TYR608-Side-OH	GLU972-Side-OE2	45.06%				**ASP613-Side-OD1**	**ARG976-Side-NH1**	**47.96%**
TYR608-Side-OH	GLU972-Side-OE1	41.74%				**ARG586-Side-NE**	**GLU972-Side-OE1**	**44.54%**
ARG586-Side-NH2	GLU972-Side-OE1	41.56%				**ARG586-Side-NH2**	**GLU972-Side-OE1**	**41.42%**
ARG586-Side-NH2	GLU972-Side-OE2	40.02%				**ASP613-Side-OD2**	**ARG976-Side-NH1**	**38.16%**
ARG586-Side-NE	GLU972-Side-OE2	39.80%				**ASP613-Side-OD1**	**ARG976-Side-NH2**	**37.14%**
PHE581-Main-N	LEU977-Side-OXT	39.72%				**PHE581-Main-N**	**LEU977-Main-O**	**35.22%**
ASP613-Side-OD1	ARG976-Side-NH1	32.02%				**ARG586-Side-NH2**	**GLU972-Side-OE2**	**34.62%**
PHE581-Main-N	LEU977-Main-O	30.64%				**ARG586-Side-NE**	**GLU972-Side-OE2**	**32.22%**
						**PHE583-Main-N**	**LEU977-Main-O**	**31.54%**

The hydrogen bonds that are formed by the same residues with those in WT system are highlighted in bold.

## Supplementary Materials

Supplementary materials can be found at http://www.mdpi.com/1422-0067/20/1/224/s1.

## Abbreviations

Shank 3	SH3 and multiple ankyrin repeat domains protein 3
SAPAP	synapse-associated protein 90/postsynaptic density-95–associated protein
MD	Molecular dynamic simulation
DCCM	Dynamic Cross-Correlation Map
PCA	Principle Component Analysis
FEL	Free Energy Landscape
MM/GBSA	Molecular Mechanics/Generalized Born Surface Area
