# Supplementary material for: Molecular Dynamics Simulations of Wild Type and Mutants of SAPAP in Complexed with Shank3
Source: Int J Mol Sci. 2019 Jan 8;20(1):224. doi: 10.3390/ijms20010224 (PMC6337207; doi:10.3390/ijms20010224)
Supplement: Supplementary file 1 [file ijms-20-00224-s001.pdf]

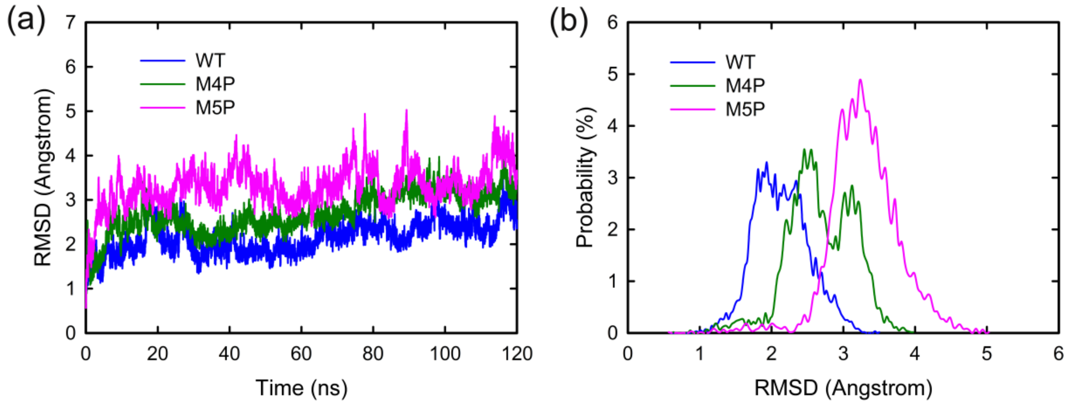

Supplementary Figure 1. (a) RMSD evolutions of protein Cα atoms with simulation time in WT, M4P and M5P systems in the duplicate MD simulations; (b) Probability distribution of RMSD values in duplicate MD simulations.

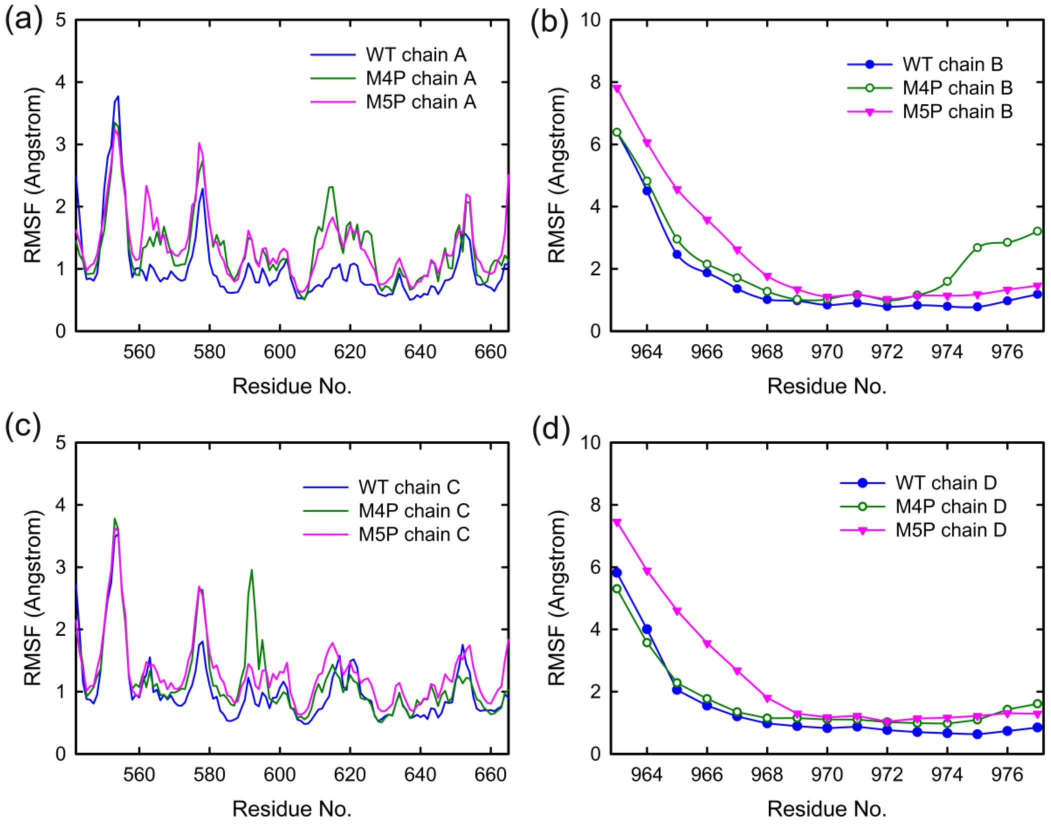

Supplementary Figure 2. Per residue RMSF of four chains in WT, M4P and M5P systems in the duplicate MD simulations. (a) RMSF of monomer A of N-PDZ (chain A); (b) RMSF of E-PBM (chain B); (c) RMSF of monomer B of N-PDZ (chain C); (d) RMSF of E-PBM (chain D).

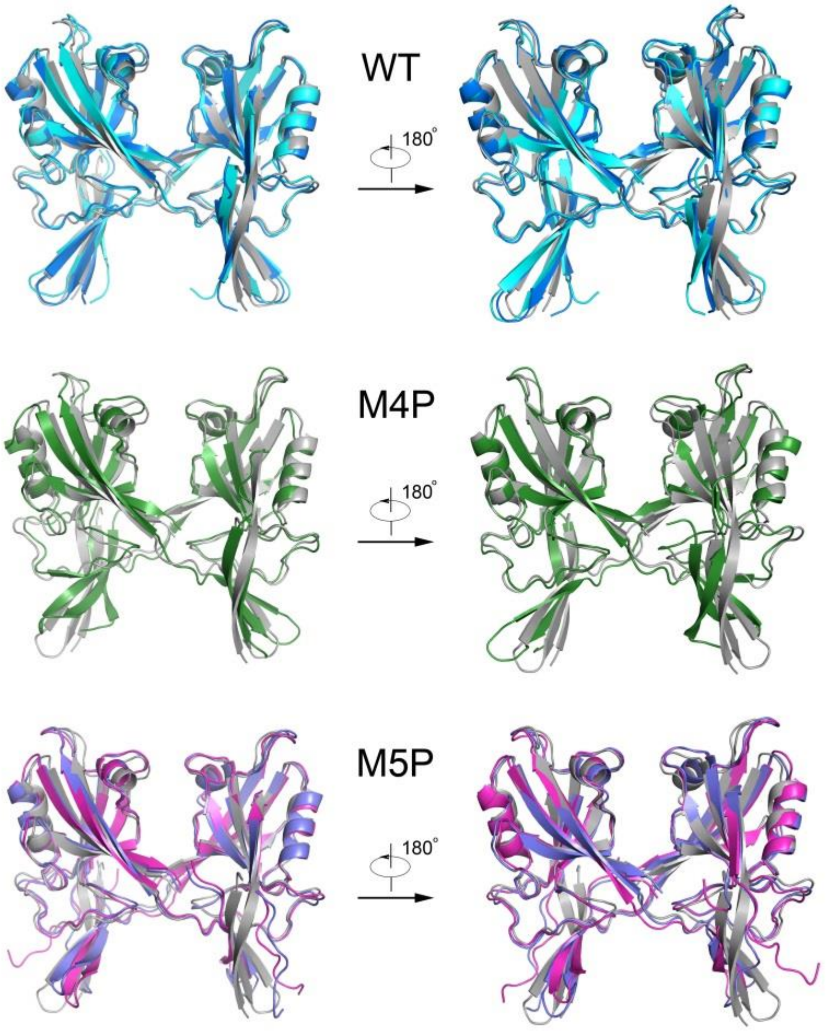

Supplementary Figure 3. Representative snapshots of WT, M4P and M5P systems. Based on the FEL analysis, local minimums are identified for three systems. Representative snapshots are extracted from the local minimums and shown in cartoon model. Conformations from different time points are shown in different colors: crystal structure – grey; snapshot from 47.36 ns in WT system (B1) - cyan; 61.32 ns in WT system (B2) – blue; 66.84 ns in M4P system (B1) – green; 63.32 ns in M5P system (B1) – magenta; 35.24 ns in M5P system (B2) – purple.

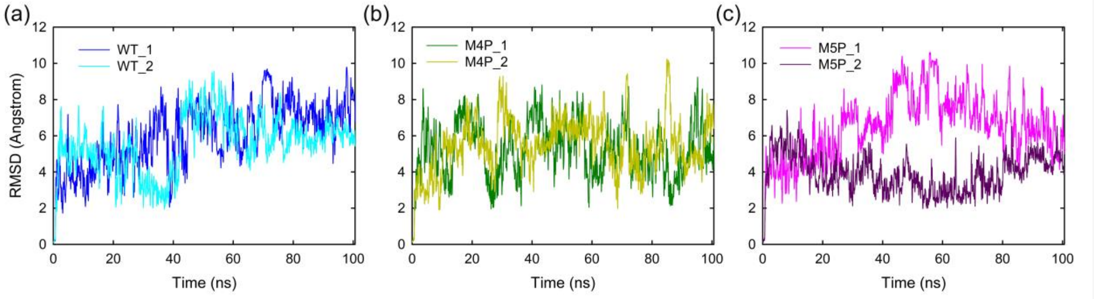

Supplementary Figure 4. RMSD evolutions of protein Cα atoms with simulation time in WT (a), M4P (b) and M5P (c) in free state MD simulations.

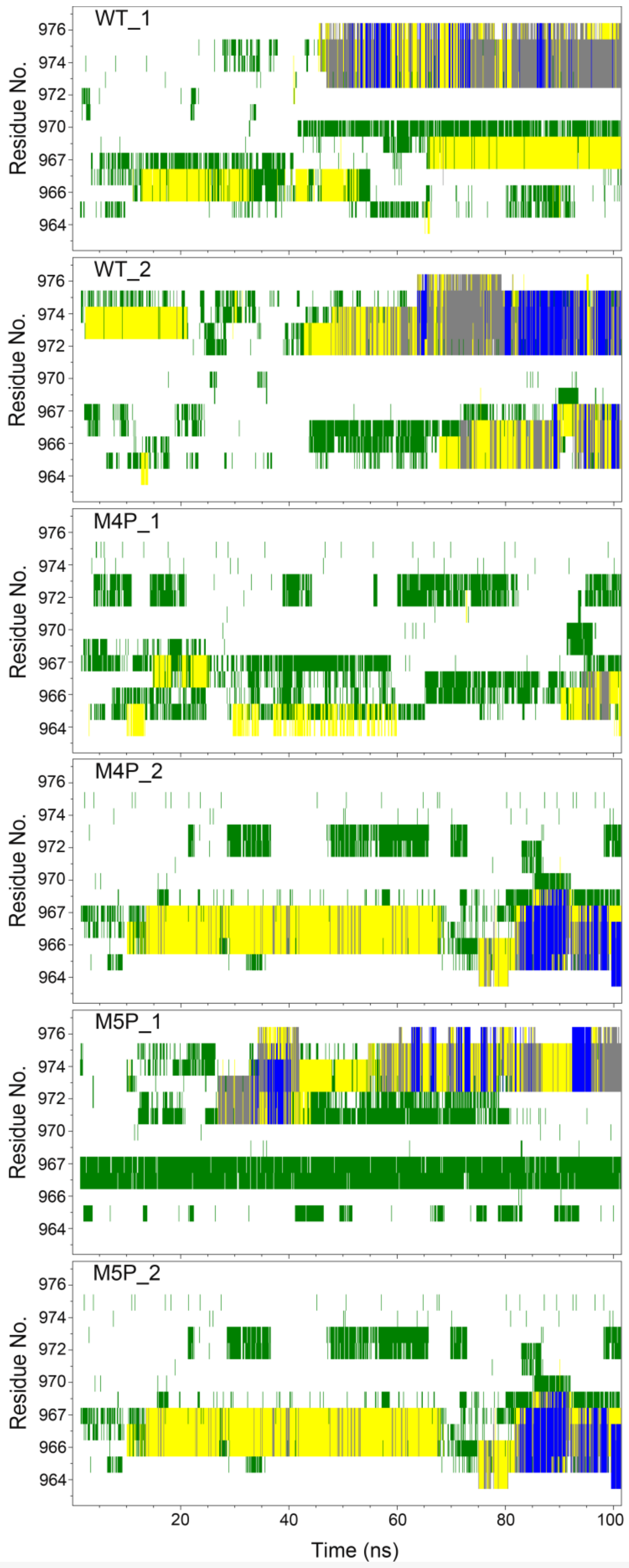

Supplementary Figure 5. Secondary structure changes versus simulation time for WT, M4P and M5P free state peptides in duplicate MD runs. Different colors denote various secondary structure: white for coil, green for bend, yellow for turn, blue for α-helix and grey for 3_{10}-helix.

Supplementary Table 1. Per residue energy decomposition for WT, M4P and M5P system. All the energy contributions are in kcal/mol and the values are shown in bold when the absolute contribution energy larger than 1 kcal/mol.

chain ID	Residue ID	Residue Name	WT	M4P	M5P
A	542	ARG	**1.36**	**1.86**	-0.17
A	543	THR	0.39	**-2.54**	-0.05
A	544	LYS	**-1.73**	**-2.17**	**-2.28**
A	545	ARG	**-9.30**	**-6.81**	**-5.47**
A	546	LEU	**-4.67**	**-4.93**	-0.31
A	547	PHE	**-5.99**	**-5.76**	**-1.30**
A	548	ARG	**-6.73**	**-7.26**	-0.01
A	549	HSD	**-4.09**	**-4.30**	-0.02
A	550	TYR	**-2.72**	**-1.42**	-0.01
A	551	THR	**-2.14**	-0.87	0.02
A	552	VAL	-0.24	0.03	0.01
A	553	GLY	-0.03	0.01	0.01
A	554	SER	0.05	0.05	0.01
A	555	TYR	0.00	0.02	-0.10
A	556	ASP	0.28	0.10	0.13
A	557	SER	-0.04	-0.12	-0.02
A	558	LEU	-0.68	-0.74	-0.44
A	559	THR	-0.16	-0.18	0.04
A	560	SER	-0.01	0.11	0.04
A	561	HSD	-0.34	-0.39	0.05
A	562	SER	-0.17	-0.12	-0.01
A	563	ASP	0.25	0.24	0.12
A	564	TYR	0.00	0.04	0.01
A	565	VAL	0.02	0.02	0.01
A	566	ILE	0.00	0.00	0.01
A	567	ASP	0.05	0.09	0.04
A	568	ASP	0.03	0.05	0.02
A	569	LYS	0.01	-0.02	0.02
A	570	VAL	0.00	0.00	0.00
A	571	ALA	0.00	0.00	0.00
A	572	ILE	0.00	0.00	0.00
A	573	LEU	0.00	0.00	0.00
A	574	GLN	0.00	0.00	0.00
A	575	LYS	0.00	-0.01	0.00
A	576	ARG	0.00	-0.01	0.00
A	577	ASP	0.00	0.00	0.00
A	578	HSD	0.00	0.00	0.00
A	579	GLU	0.00	0.01	0.00

A	580	GLY	0.00	0.00	0.00
A	581	PHE	0.00	0.00	0.00
A	582	GLY	0.00	0.00	0.00
A	583	PHE	0.00	0.00	0.00
A	584	VAL	0.00	0.00	0.00
A	585	LEU	0.00	0.00	0.00
A	586	ARG	-0.01	-0.02	-0.01
A	587	GLY	0.00	0.00	0.00
A	588	ALA	0.00	0.00	0.00
A	589	LYS	0.00	-0.01	0.00
A	590	ALA	0.00	0.00	0.00
A	591	GLU	0.00	0.00	0.00
A	592	THR	0.00	0.00	0.00
A	593	PRO	0.00	0.00	0.00
A	594	ILE	0.00	0.00	0.00
A	595	GLU	0.01	0.01	0.00
A	596	GLU	0.01	0.01	0.00
A	597	PHE	0.00	0.00	0.00
A	598	THR	0.00	0.00	0.00
A	599	PRO	0.00	0.00	0.00
A	600	THR	0.01	0.01	0.00
A	601	PRO	0.01	0.01	0.00
A	602	ALA	0.01	0.00	0.00
A	603	PHE	0.00	0.00	0.00
A	604	PRO	-0.01	-0.01	-0.01
A	605	ALA	0.00	0.00	0.00
A	606	LEU	0.00	0.00	0.00
A	607	GLN	0.00	0.00	0.00
A	608	TYR	0.00	0.00	0.00
A	609	LEU	0.01	0.00	0.00
A	610	GLU	0.02	0.02	0.01
A	611	SER	0.00	0.00	0.00
A	612	VAL	0.01	0.00	0.01
A	613	ASP	0.00	0.02	0.00
A	614	VAL	0.01	0.00	0.01
A	615	GLU	0.00	0.03	0.00
A	616	GLY	0.00	0.00	0.00
A	617	VAL	0.00	0.00	0.00
A	618	ALA	0.00	0.00	0.00
A	619	TRP	-0.01	0.00	0.00
A	620	ARG	0.00	-0.01	0.00
A	621	ALA	0.00	0.00	0.00
A	622	GLY	0.01	0.00	0.01

A	623	LEU	0.01	0.01	0.01
A	624	ARG	0.03	-0.03	0.03
A	625	THR	0.02	0.01	0.01
A	626	GLY	0.00	0.00	0.00
A	627	ASP	0.04	0.05	0.02
A	628	PHE	0.00	0.00	0.00
A	629	LEU	0.00	0.00	0.00
A	630	ILE	0.00	0.00	0.00
A	631	GLU	0.01	0.01	0.00
A	632	VAL	0.00	0.00	0.00
A	633	ASN	0.00	0.00	0.00
A	634	GLY	0.00	0.00	0.00
A	635	VAL	0.00	0.00	0.00
A	636	ASN	0.00	0.00	0.00
A	637	VAL	0.00	0.00	0.00
A	638	VAL	0.00	0.00	0.00
A	639	LYS	-0.01	-0.01	0.00
A	640	VAL	0.00	0.00	0.00
A	641	GLY	0.00	0.00	0.00
A	642	HSD	0.00	0.00	0.00
A	643	LYS	0.00	0.00	0.00
A	644	GLN	0.00	0.00	0.00
A	645	VAL	0.00	0.00	0.00
A	646	VAL	0.00	0.00	0.00
A	647	GLY	0.00	0.00	0.00
A	648	LEU	0.00	0.00	0.00
A	649	ILE	0.00	0.00	0.00
A	650	ARG	0.00	-0.01	0.00
A	651	GLN	0.00	0.00	0.00
A	652	GLY	0.00	0.00	0.00
A	653	GLY	0.00	0.00	0.00
A	654	ASN	0.00	0.00	0.00
A	655	ARG	0.00	-0.01	0.00
A	656	LEU	0.00	0.00	0.00
A	657	VAL	0.00	0.00	0.00
A	658	MET	0.00	0.00	0.00
A	659	LYS	-0.01	-0.02	0.00
A	660	VAL	0.01	0.01	0.01
A	661	VAL	0.01	0.01	0.01
A	662	SER	0.02	0.02	0.01
A	663	VAL	0.05	0.05	0.03
A	664	THR	0.07	0.11	0.07
A	665	ARG	0.06	0.08	0.08

C	542	ARG	-0.02	-0.02	-0.02
C	543	THR	0.00	0.00	0.00
C	544	LYS	-0.01	-0.01	0.00
C	545	ARG	-0.02	-0.03	-0.01
C	546	LEU	0.00	0.00	0.00
C	547	PHE	0.00	0.00	0.00
C	548	ARG	-0.02	-0.03	-0.01
C	549	HSD	0.00	0.00	0.00
C	550	TYR	0.00	0.00	0.00
C	551	THR	0.00	0.00	0.00
C	552	VAL	0.00	0.00	0.00
C	553	GLY	0.00	0.00	0.00
C	554	SER	0.00	0.00	0.00
C	555	TYR	0.00	0.00	0.00
C	556	ASP	0.01	0.01	0.00
C	557	SER	0.00	0.00	0.00
C	558	LEU	0.00	0.00	0.00
C	559	THR	0.00	0.00	0.00
C	560	SER	0.01	0.00	0.00
C	561	HSD	0.01	0.01	0.00
C	562	SER	0.01	0.00	0.00
C	563	ASP	0.06	0.07	0.02
C	564	TYR	0.00	0.00	0.00
C	565	VAL	0.01	0.01	0.01
C	566	ILE	0.01	0.00	0.01
C	567	ASP	0.02	0.03	0.01
C	568	ASP	0.04	0.03	0.03
C	569	LYS	0.12	-0.01	0.06
C	570	VAL	0.03	0.01	0.02
C	571	ALA	0.01	0.00	0.01
C	572	ILE	0.02	0.01	0.02
C	573	LEU	0.02	0.01	0.00
C	574	GLN	0.04	0.01	0.04
C	575	LYS	0.00	-0.02	-0.18
C	576	ARG	0.06	-0.02	-0.02
C	577	ASP	0.06	0.04	0.07
C	578	HSD	0.09	0.03	0.03
C	579	GLU	0.08	0.08	-0.01
C	580	GLY	**-1.79**	0.02	**-2.90**
C	581	PHE	**-2.86**	-0.02	**-3.57**
C	582	GLY	-0.99	0.00	**-1.73**
C	583	PHE	**-3.66**	0.05	**-4.29**
C	584	VAL	**-3.35**	-0.51	**-3.53**

C	585	LEU	**-4.69**	0.04	**-4.77**
C	586	ARG	**-8.88**	**-9.51**	**-8.44**
C	587	GLY	**-3.62**	-0.68	**-2.84**
C	588	ALA	**-1.79**	**-1.80**	**-1.52**
C	589	LYS	**-1.75**	**-1.23**	**-2.02**
C	590	ALA	-0.25	-0.22	-0.61
C	591	GLU	0.12	0.14	0.11
C	592	THR	0.13	0.13	0.17
C	593	PRO	0.06	0.07	0.07
C	594	ILE	-0.53	-0.34	-0.61
C	595	GLU	0.06	0.13	0.01
C	596	GLU	0.23	0.09	0.14
C	597	PHE	-0.10	-0.09	-0.12
C	598	THR	0.08	0.07	0.04
C	599	PRO	-0.02	-0.02	-0.01
C	600	THR	0.03	0.02	0.01
C	601	PRO	0.01	0.01	0.01
C	602	ALA	0.08	0.07	0.06
C	603	PHE	-0.17	-0.23	0.04
C	604	PRO	-0.03	-0.04	0.00
C	605	ALA	0.25	0.22	0.21
C	606	LEU	-0.14	-0.43	0.01
C	607	GLN	0.40	0.18	0.23
C	608	TYR	**-1.56**	**-1.86**	-0.40
C	609	LEU	0.29	0.12	0.16
C	610	GLU	-0.64	-0.25	-0.50
C	611	SER	-0.11	-0.03	-0.17
C	612	VAL	0.25	0.01	0.17
C	613	ASP	**-4.68**	0.14	**-4.36**
C	614	VAL	0.02	0.01	0.02
C	615	GLU	0.02	0.03	0.03
C	616	GLY	0.09	0.01	0.08
C	617	VAL	0.08	0.02	0.03
C	618	ALA	0.07	0.01	0.05
C	619	TRP	-0.05	-0.01	-0.03
C	620	ARG	0.03	-0.02	0.03
C	621	ALA	0.03	0.01	0.02
C	622	GLY	0.03	0.01	0.03
C	623	LEU	0.11	0.03	0.07
C	624	ARG	0.13	0.00	0.12
C	625	THR	0.14	0.06	0.04
C	626	GLY	0.04	0.04	0.08
C	627	ASP	-0.08	0.06	-0.03

C	628	PHE	-0.08	-0.06	-0.03
C	629	LEU	0.10	0.05	0.09
C	630	ILE	0.02	0.01	0.01
C	631	GLU	0.04	0.03	0.02
C	632	VAL	0.08	0.03	0.06
C	633	ASN	0.04	0.01	0.04
C	634	GLY	0.01	0.01	0.01
C	635	VAL	0.02	0.01	0.02
C	636	ASN	0.10	0.06	0.09
C	637	VAL	0.11	0.06	0.09
C	638	VAL	0.09	0.07	0.08
C	639	LYS	0.11	0.13	0.13
C	640	VAL	0.12	0.08	0.15
C	641	GLY	0.20	0.15	0.19
C	642	HSD	**-2.49**	-0.27	**-2.70**
C	643	LYS	-0.05	-0.02	-0.05
C	644	GLN	0.08	0.09	0.09
C	645	VAL	0.17	0.12	0.18
C	646	VAL	**-1.26**	-0.07	**-1.23**
C	647	GLY	-0.04	0.01	-0.03
C	648	LEU	0.13	0.04	0.12
C	649	ILE	-0.55	0.02	-0.53
C	650	ARG	-0.76	-0.04	-0.71
C	651	GLN	0.07	0.02	0.06
C	652	GLY	0.03	0.00	0.03
C	653	GLY	0.05	0.01	0.06
C	654	ASN	0.04	0.01	0.01
C	655	ARG	0.03	-0.01	0.03
C	656	LEU	-0.02	0.02	-0.05
C	657	VAL	0.02	0.01	0.01
C	658	MET	-0.01	0.01	-0.03
C	659	LYS	0.01	-0.01	0.03
C	660	VAL	0.09	0.04	0.08
C	661	VAL	0.03	0.02	0.03
C	662	SER	0.00	0.00	0.00
C	663	VAL	0.04	0.02	0.02
C	664	THR	0.01	0.00	0.00
C	665	ARG	-0.01	0.00	0.00
E-PBM	963	ALA	**2.02**	**2.36**	0.42
E-PBM	964	ASP	-0.95	-0.23	0.19
E-PBM	965	SER	**-1.64**	-0.97	0.19
E-PBM	966	ILE[a]	**-3.83**	**-2.70**	-0.53
E-PBM	967	GLU[a]	**-2.90**	**-3.26**	-0.34

E-PBM	968	ILE[a]	**-4.61**	**-4.44**	0.22
E-PBM	969	TYR[a]	**-3.78**	**-4.73**	**-1.91**
E-PBM	970	ILE[a]	**-5.61**	**-5.90**	**-4.34**
E-PBM	971	PRO	**-4.04**	**-3.99**	**-4.83**
E-PBM	972	GLU	**-9.68**	**-10.74**	**-8.31**
E-PBM	973	ALA	**-4.15**	-0.81	**-4.45**
E-PBM	974	GLN[b]	**-3.66**	**-3.37**	**-1.98**
E-PBM	975	THR[b]	**-5.28**	**-2.59**	**-5.50**
E-PBM	976	ARG[b]	**-8.22**	-0.29	**-7.18**
E-PBM	977	LEU[b]	**-3.49**	0.37	**-4.12**

[a]: Residues marked with "a" are mutated to Pro in M5P system.

[b]: Residues marked with "b" are mutated to Pro in M4P system.
